# Acute Myocarditis Following Dupilumab Initiation

**DOI:** 10.7759/cureus.77797

**Published:** 2025-01-21

**Authors:** Samrita Naidu, Rani Vatti

**Affiliations:** 1 Allergy and Immunology, Rio Americano High School, Sacramento, USA; 2 Allergy and Immunology, Kaiser Permanente, Sacramento, USA

**Keywords:** acute eosinophilic myocarditis, acute hypersensitivity myocarditis, acute myocarditis, dupilumab, dupixent, side effects

## Abstract

A 48-year-old male patient developed an acute rash, fever, myalgia, abdominal pain, tachycardia, near syncope, and chest pain after receiving one dose of dupilumab for chronic rhinosinusitis with nasal polyps (CRSwNP). He presented with hypotension, elevated troponin, hypereosinophilia, ST elevation with T wave abnormalities, and a mildly dilated left ventricle with severely reduced systolic function. He was diagnosed with acute myocarditis. He was treated with high-dose steroids, which rapidly alleviated his symptoms and led to a substantial improvement in his heart function.

## Introduction

Myocarditis is an inflammatory condition of the cardiac muscle caused by a viral infection or secondary to inflammation from a bacterial, parasitic, or fungal pathogen. In rare instances, it can result from a hypersensitivity allergic reaction to an inciting drug, a condition known as hypersensitivity myocarditis. Hypersensitivity myocarditis, a form of eosinophilic myocarditis, is typically characterized by acute rash, fever, peripheral eosinophilia, tachycardia, and electrocardiographic (EKG) abnormalities, including ST elevation [1]. Hypersensitivity myocarditis is treated by discontinuing the offending drug and initiating corticosteroid therapy. Dupilumab is a human monoclonal IgG4 antibody that inhibits interleukin-4 (IL-4) and interleukin-13 (IL-13) signaling by binding to the IL-4 receptor α subunit (IL-4Rα), reducing type 2 helper T-cell-mediated inflammation. Dupilumab effectively treats type 2 inflammatory diseases such as atopic dermatitis, asthma, and chronic rhinosinusitis with nasal polyps (CRSwNP) [2]. Common adverse reactions to this medication include injection site reactions, conjunctivitis, keratitis, nasopharyngitis, and facial redness [3]. Dupilumab is known to cause hypereosinophilia [4]. Here, we present a case of acute myocarditis after the initiation of dupilumab.

## Case presentation

A 48-year-old Caucasian male patient with a history of mild allergic rhinitis without any childhood history of asthma was referred to an allergist by his otolaryngologist for CRSwNP. He had a six-month history of nasal polyps with congestion and yellow nasal discharge despite multiple courses of prednisone and antibiotics. He had mild intermittent wheezing, so he was also diagnosed with mild adult-onset asthma. His pulmonary function test was normal with FVC (forced vital capacity) of 93%, FEV1 (forced expiratory volume in one second) of 93%, and FEV1/FVC ratio of 79. His FENO (fractional exhaled nitric oxide) was markedly elevated at 91. He had eosinophilia with an absolute eosinophil count (AEC) of 1200/μL (0-400/μL). He had insignificant allergy sensitization. He did not have atopic dermatitis or dysphagia.

The genetic evaluation for familial eosinophilia syndrome showed no identifiable pathogenic mutations. Chromosome analysis by FISH (fluorescence in situ hybridization) was negative for *FIP1L1::PDGFRA* fusion, *PDGFRB* rearrangement, and *FGFR1* rearrangement. Next-generation sequencing (NGS) did not reveal mutations typically associated with myelodysplasia or leukemia. CT sinuses showed sinusitis with polyps. Functional endoscopic sinus surgery with nasal septoplasty and biopsy of bilateral sinus contents showed chronic inflammation and edema of polypoid respiratory mucosa. His symptoms returned about two weeks after surgery. He was using budesonide sinus rinses and prednisone. He had a recurrence of nasal polyps within two months of surgery, so he was advised to start dupilumab 300 mg subcutaneously every two weeks. He was also on a prednisone taper, taking 20 mg of prednisone daily at that time.

**Figure 1:**
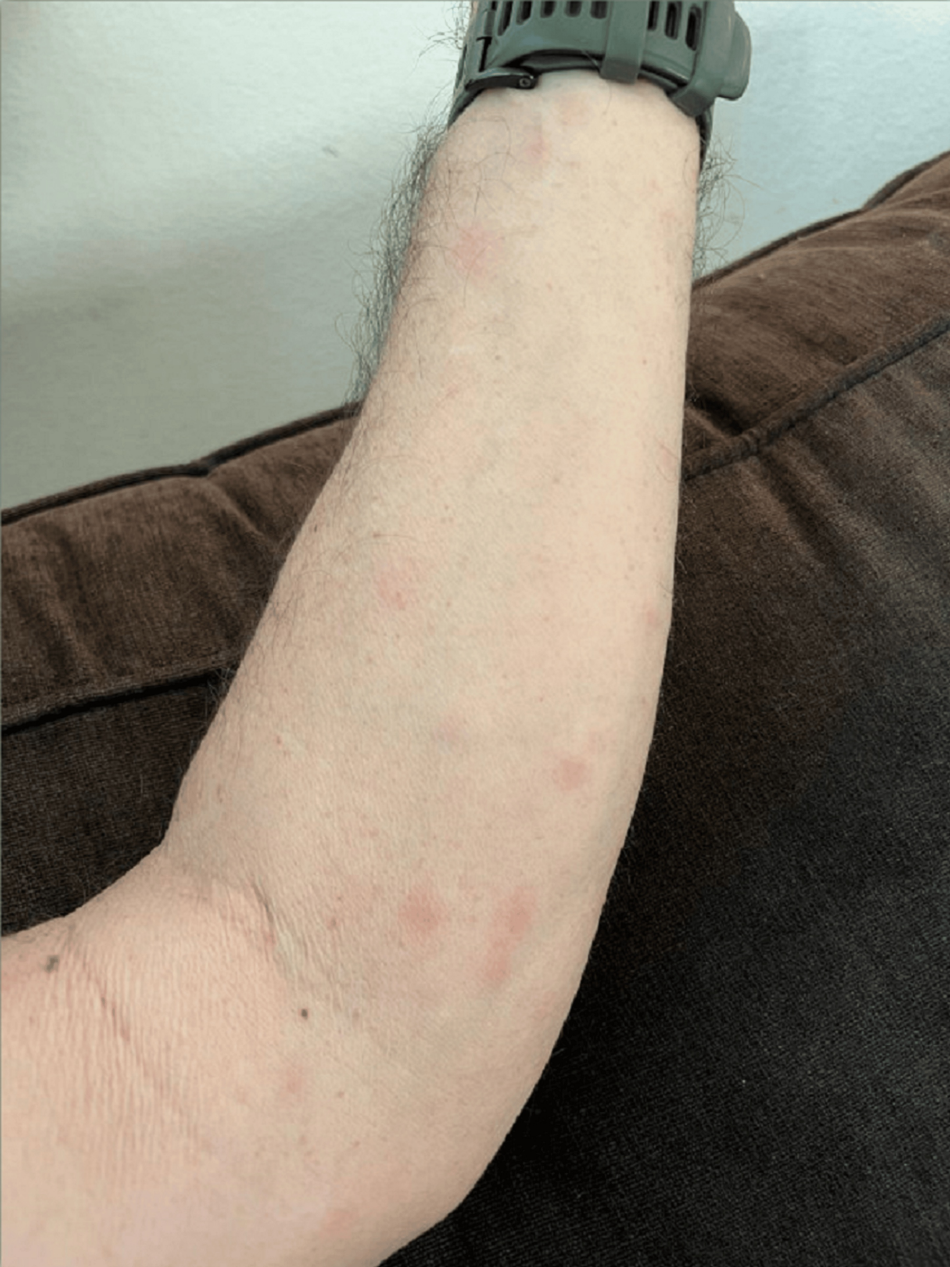
Erythematous maculopapular rash on the forearm.

The day after his first injection of dupilumab, he developed a rash, bilateral leg pain, fever, chills, and tachycardia. His rash was non-itchy, non-burning, erythematous, and maculopapular. The rash initially appeared on his forearms (Figure 1), spread to his shoulders and underarms, and then resolved spontaneously within three to four days. Four days later, he also had left upper quadrant abdominal pain, malaise, and near syncope. He stated that he stopped taking his prednisone 10 mg due to abdominal pain. His CT angiogram was negative and did not show pulmonary embolism. CT of the head was negative for any intracranial process. On day 10, he was hospitalized for chest pain, significant fatigue, myalgia, hypotension, and a fever of 101℉ with night sweats. He had left-sided chest pain radiating to his left shoulder, which increased with leaning forward and deep breathing. His review of systems was negative for any other history of rash, skin lesions, joint pain, joint swelling, morning stiffness, numbness, tingling, focal weakness, pneumonia, lymphadenopathy, blood clots, or diarrhea.

**Figure 2:**
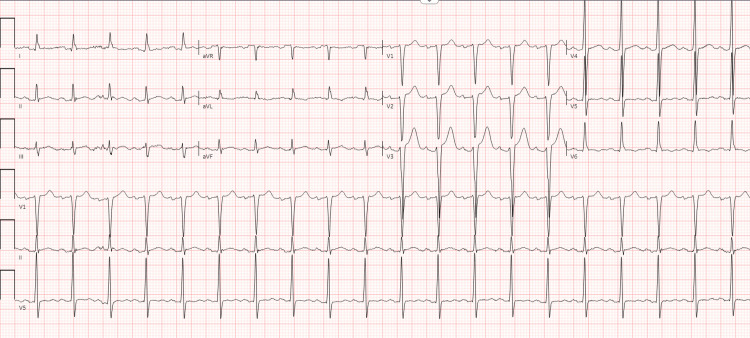
Electrocardiography showing sinus tachycardia and ST elevation in V2-V3 with non-specific T wave abnormalities.

He had elevated troponin I of 2417 ng/L (< 20 ng/L), hypereosinophilia with an AEC of 9360/μL (0-400/μL), elevated erythrocyte sedimentation rate of 99 mm/hour (0-15 mm/hour), elevated C-reactive protein of 22.2 mg/dL (≤ 0.9 mg/dL), normal transaminases, normal urine analysis, and normal creatinine. A complete blood count showed an elevated white cell count of 20.7 K/μL (3.7-11.1 K/μL), otherwise normal. His anti-neutrophil cytoplasmic antibody test showed that the myeloperoxidase antibody was positive at 7 (≤ 0.9). EKG showed sinus tachycardia and ST elevation in V2-V3 with non-specific T wave abnormalities (Figure 2). Echocardiography revealed a mildly dilated left ventricle with diffuse hypokinesis without any thrombus and severely reduced systolic function with an estimated ejection fraction of 25 to 30% (Videos 1-3). Chest X-rays showed trace pleural effusion without any infiltrates. CT angiograms of the head and neck were normal.

**Video 1:** This is a standard view of echocardiography showing a severely reduced left ventricular systolic function with diffuse hypokinesis and an estimated ejection fraction of 25 to 30%.

**Video 2:** This is also a standard view of echocardiography showing a severely reduced left ventricular systolic function with diffuse hypokinesis and an estimated ejection fraction of 25 to 30%.

**Video 3:** This echocardiography shows a contrast view, confirming the absence of left ventricular apical thrombus.

Based on his clinical presentation, including acute chest pain, fever, fatigue, palpitations, and hypotension, along with EKG findings, elevated troponin levels, hypereosinophilia, and echocardiographic results, the cardiology team was certain about the diagnosis of acute eosinophilic myocarditis. They determined that further diagnostic tests, such as a cardiac MRI or endomyocardial biopsy, would not change the management approach, and therefore, an endocardial biopsy was not performed. He was treated with intravenous methylprednisolone for two to three days. His dupilumab was permanently discontinued. He was discharged home on 60 mg oral prednisone daily, which was gradually tapered as an outpatient. His symptoms were completely resolved with high-dose corticosteroid treatment. His repeat echocardiography four weeks later showed normal left ventricle size with an improved estimated systolic ejection fraction of 50 to 55%. As his nasal symptoms recurred with a reduction in prednisone dose below 20 mg, he was started on mepolizumab 100 mg, which effectively alleviated both his symptoms and eosinophilia, facilitating a successful tapering of prednisone.

## Discussion

Dupilumab, a monoclonal antibody targeting IL-4Rα, inhibits the signaling of cytokines IL-4 and IL-13, two pivotal drivers of type 2 inflammation that are important in diseases such as CRSwNP and asthma. It has also been approved to treat atopic dermatitis, eosinophilic esophagitis, and prurigo nodularis [2]. In addition to the common adverse reactions previously mentioned, other potential side effects of dupilumab include alopecia areata, psoriasis, cutaneous T-cell lymphoma, arthritis, and skin peeling [3]. Dupilumab is also known to cause transient eosinophilia and hypereosinophilia [4]. The increase in blood eosinophils is likely due to the inhibition of endothelial VCAM-1 and ICAM-1 expression, which is essential for eosinophil extravasation and dependent on IL-4Rα [5]. Dupilumab-induced hypereosinophilia can, in turn, be associated with severe adverse effects such as eosinophilic myocarditis. Hypereosinophilia with severe adverse effects has been reported within the first several weeks of starting dupilumab therapy [5,6]. Additionally, eosinophilic granulomatosis with polyangiitis (EGPA) has been reported after treatment with dupilumab [7]. Although EGPA cannot be completely ruled out, our patient was not initially considered to have systemic vasculitis, such as EGPA, based on his clinical presentation, which showed no evidence of involvement in other organs, including the skin (no vasculitic rash or other skin lesions), lungs, kidneys, or peripheral nerves.

Hypersensitivity myocarditis is a drug-related form of eosinophilic myocarditis in which an autoimmune reaction in the heart causes myocardial inflammation. Hypersensitivity myocarditis can be potentially fatal. In a retrospective study from a pathology institute, in 20 out of 24 patients with drug-induced hypersensitivity myocarditis, the primary cause of death was hypersensitivity myocarditis [1]. Due to the potentially fatal nature of this condition, early recognition, timely diagnosis, and prompt initiation of steroid treatment are critical to improving patient outcomes.

Given that our patient's symptoms began one day after receiving a dose of dupilumab, progressively worsened, and ultimately resulted in a diagnosis of acute myocarditis, we suspect a relationship with dupilumab. The patient's discontinuation of prednisone due to abdominal pain a few days prior to hospitalization could have contributed to his hypereosinophilia and acute eosinophilic myocarditis. Further studies, case reports, and the development of biomarkers are needed to better understand any association with the medication and its underlying mechanisms. To our knowledge, this is the first reported case of this scenario in the literature.

## Conclusions

Careful management is essential when tapering prednisone in patients with eosinophilic inflammation, especially when starting eosinophilic modulators such as dupilumab, which can cause transient hypereosinophilia. This could either signal an adverse drug reaction or falsely suggest a worsening of the underlying condition, leading to unnecessary discontinuation of effective treatment. Nonspecific cardiac findings (electrocardiographic changes, tachycardia, or serum enzyme elevations) in patients receiving any drug associated with hypersensitivity should raise suspicion for drug-related hypersensitivity myocarditis. Nonspecific cardiac findings with hypereosinophilia should raise concern for acute eosinophilic myocarditis. Early recognition, diagnosis, and prompt initiation of steroid treatment are important for the effective management of this condition. Proper timing of prednisone taper and dupilumab initiation, combined with close monitoring, is key to distinguishing between these potential complications and ensuring optimal care.
